# Network meta-analysis of short-term effects of different strategies in the conservative treatment of AIS

**DOI:** 10.1186/s40001-021-00526-6

**Published:** 2021-06-13

**Authors:** Kepeng Li, Jun Miao, Jingan Zhang

**Affiliations:** 1 Second Central Hospital of Baoding, 57 Fan Yang Middle Road, Zhuozhou, Baoding, Hebei China; 2 Tianjin Hospital, 406 Jiefang South Road, Hexi District, Tianjin, China

**Keywords:** Adolescent idiopathic scoliosis, Brace, Scoliosis-specific exercises

## Abstract

**Purpose:**

To evaluate the short-term effects of different conservative treatments on in adolescent idiopathic scoliosis.

**Methods:**

By searching the relevant literature of adolescent idiopathic scoliosis, the curative effects of the three regimens of bracing therapy combined with scoliosis-specific exercises, simple treatment with brace and simple scoliosis-specific exercises were compared. Review manager 5.3, Stata MP16 and Network software packages were used for Reticular Meta-analysis of Cobb’s angles before and after treatment.

**Results:**

A total of 364 patients were included in four clinical studies. Reticular meta-analysis showed that the short-term effect of bracing treatment combined with scoliosis-specific exercises was better than that of treatment with brace and scoliosis-specific exercises, with effects of 2.71(95% CI 0.83–4.58) and 3.67(95% CI 1.21–6.14), respectively. There was no statistical difference between simple bracing therapy and scoliosis-specific exercises.

**Conclusion:**

Among the three common conservative treatments of adolescent idiopathic scoliosis, the short-term effect of bracing treatment combined with scoliosis-specific exercises is better than that of bracing treatment or scoliosis-specific exercises.

## Background

Adolescent idiopathic scoliosis (AIS) is an unexplained three-dimensional malformation of the spine, with an incidence of about 2–3% in the population between 10 years of age and bone maturation [1]. AIS can lead to many problems such as limited function, pain, poor appearance and decreased quality of life [2, 3]. The most commonly used method for measuring the severity of scoliosis is the Cobb’s angle, which is mainly used to measure the lateral angle of the spine on the frontal surface using standard anterior and posterior X radiographs [4]. Bracing therapy is a classic means for AIS’s conservative treatment to prevent scoliosis angle from increasing. Correct scoliosis by wearing braces so that scoliosis does not progress over time [5]. In addition to the living inconvenience and psychological pressure caused by the treatment of brace, there are also defects in the correction of three-dimensional malformation and aggravation of flat back deformity [6]. Therefore, people are trying to find a more proactive way to treat AIS.

Scoliosis-specific exercise (SSE) is commonly used to treat patients with mild to moderate scoliosis and consists of a set of scoliosis-specific exercise regimens, which is adjusted according to the patient’s personalized scoliosis site [7], Cobb’s angle, and clinical characteristics. SSE is mainly including three-dimensional active correction, daily life integration, stable correction posture, and patient education. SSE is divided into various schools, such as Schroth, SEAS, lateral shift, etc. The aim of SSE is reducing the angle of scoliosis and preventing its progression and stabilizing the therapeutic effect [8].

So far, although both bracing therapy and SSE have become important means of conservative treatment of AIS, there is no systematic comparative study on the effects of different treatment options. A number of previous small sample studies have demonstrated better efficacy existing in treatment combining SSE with bracing, but the results remains controversial. To make clear the therapeutic effect of these three treatment schemes on AIS, the short-term curative effect of the treatment AIS is analyzed by net meta, which will provide a reference for the choice of treatment plan of mild to moderate AIS.

## Materials and methods

### Search strategy

The following computerised bibliographic databases were searched: PubMed, Ovid database, Cochrane library, Embase, and Google academic. Stepwise documentation of the entire search process was implemented. The publication time range of study was 1990–October 30, 2020. The conduction date of search was October 30, 2020.

The search queries was: AIS and (exercise therapy or PSSE or Schroth or SEAS or rehabilitation) and (orthosis or brace). Two authors were assigned to search database independently using the same strategy. Based on the inclusion and exclusion criteria, the authors independently reviewed the titles, abstracts and full-text articles retrieved in the initial search. Disagreements of the authors regarding accepting full-text articles were discussed until consensus was achieved.

### Study inclusion and exclusion criteria

Inclusion criteria: (1) related study of AIS brace and SSE treatment; (2) inclusion of patient Cobb’s angle  >  10°; (3) prospective cohort design; (4) outcome variable included Cobb’s angle.

Exclusion criteria: (1) the way of exercise is the study of popular sports, recreational activities and general physiotherapy. (2) Study was conducted in patients with non-specific scoliosis (congenital, neurological, post-traumatic). (3) Did not detail the options of SSE treatment.

### Data extraction

The data were extracted and captured on a Microsoft Excel spreadsheet by one author to ensure continuity. Data extracted from the articles included the following categories:

method of random sequence generation, blind method in result measurement, description of missing data; patient inclusion criteria, follow-up time; data of the time, frequency, type, duration of SSE intervention; outcome measures. Three key criteria of randomization, allocation concealment and blindness in outcome measures published by the Cochrane team was used to evaluate Bias risk. The research team members cross-checked the information independently and the results of evaluation was compared to reach consensus.

### Data analysis

The bias of the article was assessed using Revman5.3 software (Cochrane IMS). The effect variables used for merging were the mean of Cobb’s angular difference (MD) and the standard deviation (SD) of difference before and after treatment. The Stata MP 16 and Network software package were used to analyze the Cobb’s angle. First, the mesh diagram is made, and the inconsistency model is used to test consistency. If the *P* value is greater than 0.05, then the consistency model is used to analyze the data and make the forest map. The node splitting method is used to test the local inconsistency.

## Results

### Studies

**Fig. 1:**
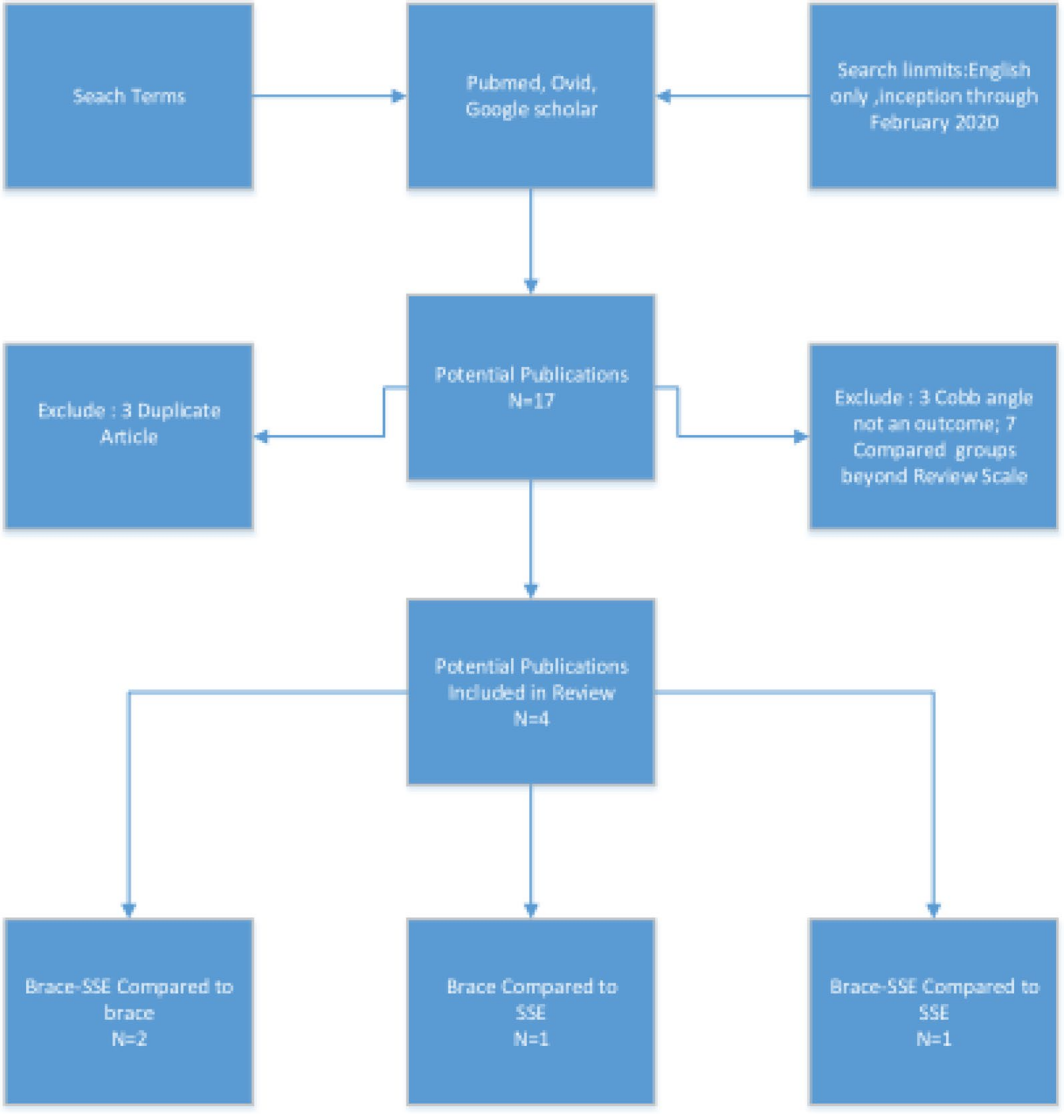
Flow chart of the study

According to the predetermined search strategy, 412 related documents were retrieved, including 407 in English and 5 in Chinese. The repeated literature, review, medical record report and experimental design literature were excluded by reading the title, leaving 55 articles. After reading the abstract and full text, the papers which not met the inclusion criteria were excluded in terms of the subjects and interventions. Finally four papers were included in study [9–12], including a total of 364 patients. The search strategy and results are illustrated in Fig. 1.

### Description of study sample

**Fig. 2:**
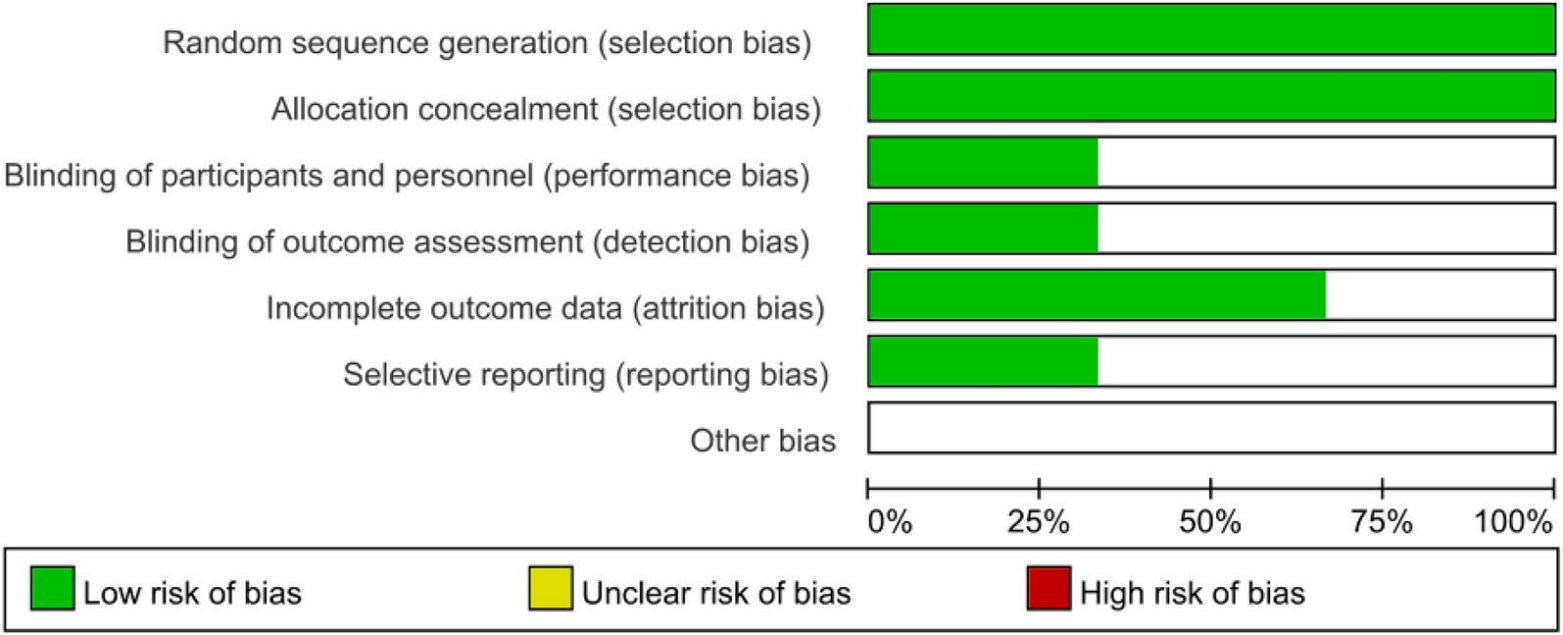
Risk of bias for included studies

Table 1 summarises the sample descriptions and interventions of the four included papers. All papers had small sample sizes, contributing to a total sample size of 364 participants, consisting of 56 in the bracing-SSE combined groups, 71 in the bracing groups and 39 in the SSE groups. All these SSE Schools have same aim and achieve them in the same order and time. The mean age of the participants among the studies ranged between 11.8 and 13.5 years. All the participants were diagnosed with AIS and were otherwise healthy. Bias evaluation of the four studies included are shown in Fig. 2.

**Table 1:** Summary of the four included papers

Study	Time (w)	N	Sex (f)	Age (y)	Inclusion criteria (Cobb’s)	SSE	brace
Schreiber [10] (randomized control)	24				Age (10–18)	Schroth	
					Cob (10˚–45˚)	1/d	
1 Brace-SSE		25	23	13.5			
2 Brace		25	23	13.3			
Zheng [11] (randomized control)	24				Age (10–17)	SEAS	TLSO
					Cob (10˚–40˚)	1/d	
3 SSE		29	22	12.4			
2 Brace		24	19	12.3			
Gao [9] (randomized control)	24				Age (10–1 year postmenstruation)	SEAS	TLSO
					Cob (25˚–40˚)	1/d	
1 Brace-SS		23	18	12.2			
2 Brace		22	18	12.1			
Sayyad [12] (cohort)	12				Age (6–16)	1/d	Milwaukee
					Cob (15˚–45˚)		
1 Brace-SSE		8	4	12.1			
3 SSE		10	6	11.8

### Results of net meta analyses

**Fig. 3:**
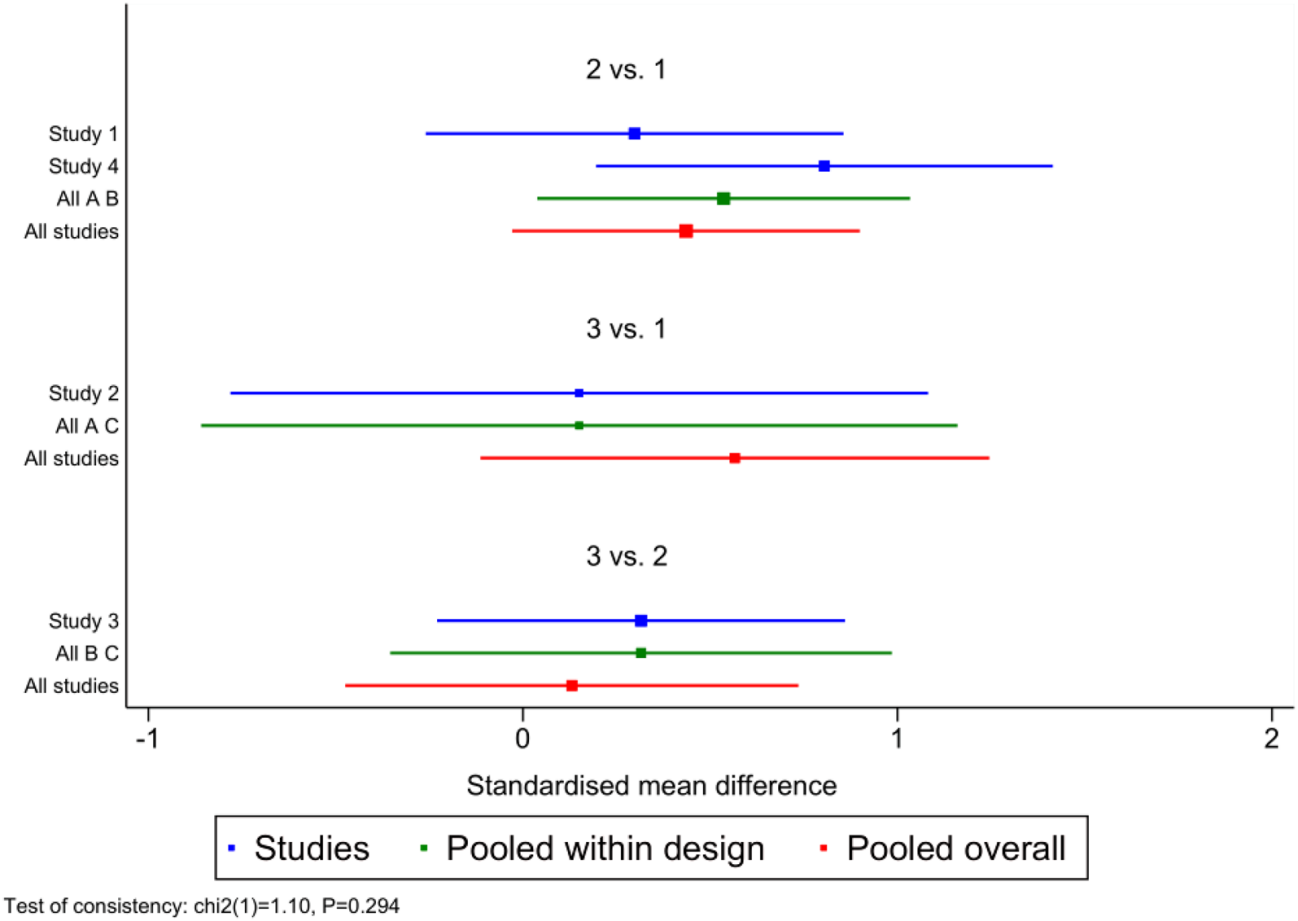
Forest plot of the three groups. 1 **A**: Bracing-SSE combined groups; 2 **B**: bracing groups; 3 **C**: SSE groups

The inconsistency model test was not significant ($\chi$^2^  =  0.68, *P*  =  0.41), the consistency model can be used for data analysis. The forest plot showed that the Cobb’s angle improvement in the brace-SSE combined groups is better than that of the other groups (Fig. 3).

#### Local inconsistency test

There is no local inconsistency existed (Table 2).

**Table 2:** Results of local inconsistency tests

Side	Direct		Indirect		Difference		
	Coef.	Std. Err.	Coef.	Std. Err.	Coef.	Std. Err.	*P* > |*z*|
A B	2.932	0.995	− 0.040	3.476	2.972	3.616	0.411
A C	1.120	3.351	4.092	1.357	− 2.972	3.616	0.411
B C	1.160	0.922	− 1.812	3.496	2.972	3.616	0.411

#### The comparative results

**Fig. 4:**
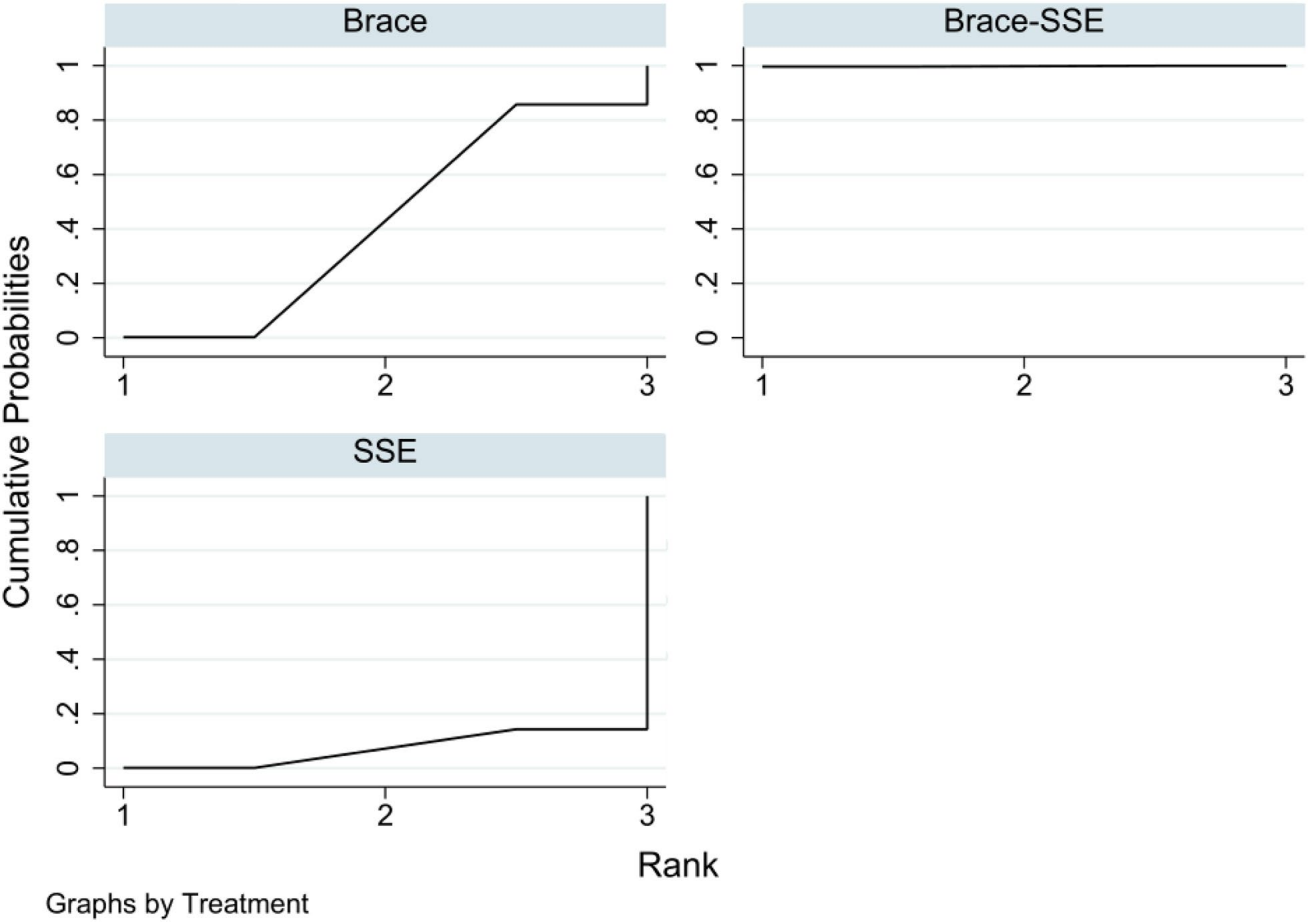
Treatment Relative Ranking

**Fig. 5:**
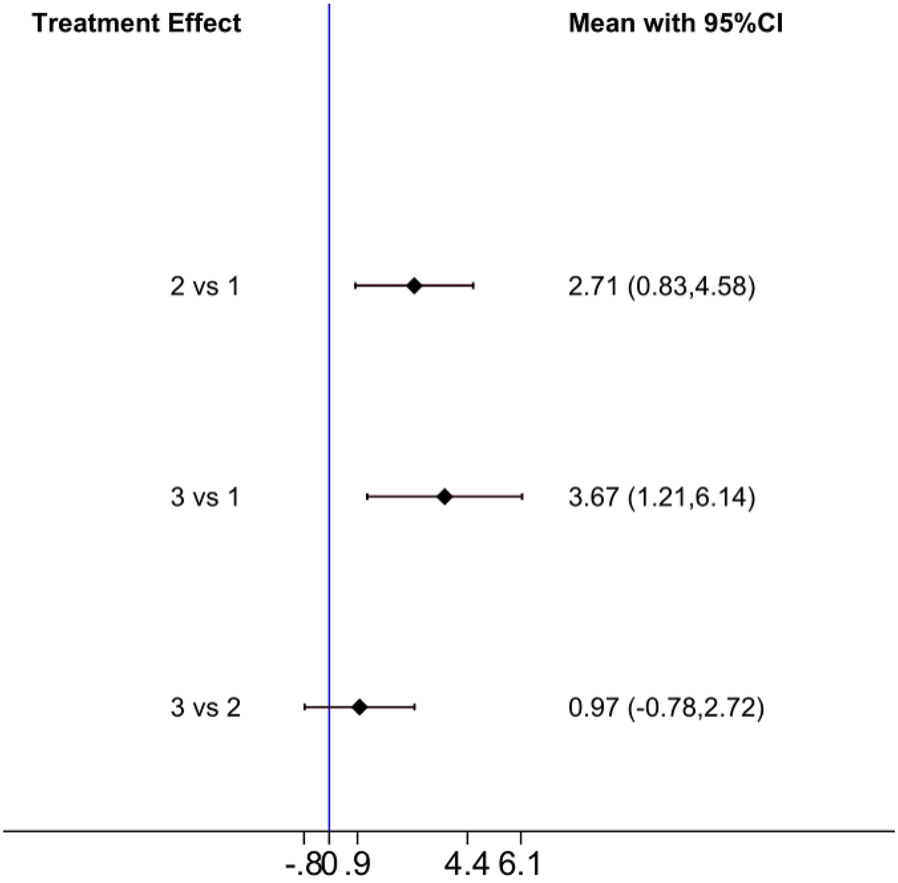
Comparative forest plot

The league table and comparative forest plot showed that the correction effect of bracing-SSE combined groups was better than that of simple bracing or SSE groups. Whereas there was no statistical difference between bracing and SSE groups. Table 3; Fig. 4 show the relative ranking results, whereas Table 4; Fig. 5 show the statistical differences among three groups.

**Table 3:** Treatment Relative Ranking

Treatment	SUCRA	PrBest	Mean rank
Brace-SSE	99.8	99.6	1.0
Brace	43.0	0.2	2.1
SSE	7.2	0.1	2.9

**Table 4:** League table

Brace-SSE	Brace	SSE
Brace-SSE	2.71 (0.83, 4.58)	3.67 (1.21, 6.14)
− 2.71 (− 4.58, − 0.83)	Brace	0.97 (− 0.78, 2.72)
− 3.67 (− 6.14, − 1.21)	− 0.97 (− 2.72, 0.78)	SSE

## Discussion

Brace and SSE therapy are both important means of AIS conservative treatment. The theoretical basis of bracing therapy is the application of external force to restore the spine in the malformed state to the physiological state, so that the spine can continue to grow in the physiological position, thus prevent the spinal deformity from progression [13]. Rehabilitation physicians should be part of the multidisciplinary team in AIS conservative treatment [14]. The main goal of the additional SSE exercise for bracing treatment is to eliminate or reduce the side effects (flat back deformity, disuse muscle atrophy) caused by bracing treatment. In fact, the sponsors of SSE are not intended to replace brace treatment, but to be used in combination with brace as an auxiliary intervention, though SSE alone may temporarily reduce the Cobb’s angle of AIS [15].

For the outcomes, the net meta-analyses showed that brace-SSE results in greater improvement of spinal deformity at follow-up (Cobb angle) in comparison with pure brace or SSE interventions. The time-dependent passive mechanism of bracing therapy combined with active correction mechanism can bring more benefits to patients. Previous controlled studies on the curative effect between brace and SSE showed that the outcomes of brace treatment was better than that of SSE [16, 17]. Schreiber et al. [18] observed that adding SSE might address a need and offer a treatment complement in patients who are not fully compliant with brace treatment. Consistently with previous studies, our outcomes suggested that adding SSE to bracing would lead to better outcomes, as compared to bracing alone. However, there was no statistical difference found between brace and SSE in this net meta. The difference of results may exist in the short follow-up time of included study. Due to the time-dependent passive correction mechanisms, the advantages of brace will not be spotted in a short 24-week period.

SSE consist of a program of curve-specific exercise protocols which are individually adapted to a patients’ curve site, magnitude and clinical characteristics [19]. There are several schools under the SSE banner focused on the treatment of AIS, each of the schools promotes a unique technique and unique exercises. However, the methods’ overall goals are the same, as each method seeks to treat all the 3D scoliosis deformity by realigning the spine, rib cage, shoulders and pelvis to ‘normal’ anatomical postures [20]. Many professionals of AIS are usually not clear the different value between bracing treatment and SSE: while some experts overestimate the importance of SSE, The other part underestimates its importance. The evidence about the effectiveness of SSE is growing, with more high quality research studies being published in recent years. As the results of our net meta research displayed, AIS patients can take maximum benefits only when the brace-SSE treatment take place. The results are consistent with previous studies.

## Limitations

Although only four papers were included in this mesh meta, the included population and intervention characteristics of the literature were similar, comparable and transferable. With the prolongation of interventional time, the interference of compliance factors on the results increased. The study reasonably controlled the influence of confounding factors on the outcome judgment by including papers in which the interventional time are about 24 weeks.

## Conclusion

For conservative treatment of AIS, the combination of bracing and SSE treatment is significantly better than single brace or SSE treatment. Clinical AIS treatment should include the team collaboration of rehabilitation physicians, clinicians.

## Data Availability

Data available within the article or its supplementary materials.
